# Influence of verapamil on the pharmacokinetics of oridonin in rats

**DOI:** 10.1080/13880209.2019.1688844

**Published:** 2019-11-21

**Authors:** Jing Liu, Ning Zhang, Na Li, Xiaocheng Fan, Ying Li

**Affiliations:** a Department of Pediatric Medicine, Yidu Central Hospital of Weifang, Shandong, China;; b Department of Neonatology, Yidu Central Hospital of Weifang, Shandong, China;; c Department of Oncology, Jining Traditional Chinese Medicine Hospital, Jining, China

**Keywords:** CYP3A4, *P-gp*, drug–drug interaction

## Abstract

**Context:** Oridonin has been traditionally used in Chinese treatment of various cancers, but its poor bioavailability limits its therapeutic uses. Verapamil can enhance the absorption of some drugs with poor oral bioavailability. Whether verapamil can enhance the bioavailability of oridonin is still unclear.

**Objective:** This study investigated the effect of verapamil on the pharmacokinetics of oridonin in rats and clarified its main mechanism.

**Materials and methods:** The pharmacokinetic profiles of oral administration of oridonin (20 mg/kg) in Sprague-Dawley rats with two groups of six animals each, with or without pre-treatment of verapamil (10 mg/kg/day for 7 days) were investigated. The effects of verapamil on the transport and metabolic stability of oridonin were also investigated using Caco-2 cell transwell model and rat liver microsomes.

**Results:** The results showed that verapamil could significantly increase the peak plasma concentration (from 146.9 ± 10.17 to 193.97 ± 10.53 ng/mL), and decrease the oral clearance (from 14.69 ± 4.42 to 8.09 ± 3.03 L/h/kg) of oridonin. The Caco-2 cell transwell experiments indicated that verapamil could decrease the efflux ratio of oridonin from 1.67 to 1.15, and the intrinsic clearance rate of oridonin was decreased by the pre-treatment with verapamil (40.06 ± 2.5 vs. 36.09 ± 3.7 µL/min/mg protein).

**Discussion and conclusions:** These results indicated that verapamil could significantly change the pharmacokinetic profile of oridonin in rats, and it might exert these effects through increasing the absorption of oridonin by inhibiting the activity of *P-gp*, or through inhibiting the metabolism of oridonin in rat liver. In addition, the potential drug–drug interaction should be given special attention when verapamil is used with oridonin. Also, the dose of oridonin should be carefully selected in the clinic.

## Introduction

Oridonin is a diterpenoid extracted from *Rabdosia rubescens* (Hemsl.) Hara (Labiatae), which is traditionally used in China for the treatment of tonsillitis and a variety of cancers. Oridonin injection is used alone or in combination with other drugs to treat human cancers (Xu et al. 2006). It has reported that oridonin can inhibit the metastasis of human ovarian cancer cells, colorectal cancer cells and it can inhibit human pancreatic cancer and lung cancer (Gui et al. 2017; Yao et al. 2017; Park et al. 2018; Yang et al. 2018; Wang and Zhu 2019). Moreover, oridonin performed synergistic antiproliferative and apoptosis-inducing effects on human myeloid leukaemia cells, when combined with valproic acid (Li and Ma 2019). However, the therapeutic uses of this compound are limited by poor bioavailability. Only 11% was available after oral administration in rats (Xu et al. 2006).

The poor bioavailability can be attributed to many factors, including the low solubility and dissolution rate, poor permeability in intestine, and fast elimination rate (He et al. 2015; Zhang et al. 2015). Verapamil was developed as a calcium channel blocker to treat hypertension, and it has been reported as a inhibitor of *P-gp* and some CYP enzymes (Piao et al. 2008; Zhu et al. 2017; Chen et al. 2018), therefore, the absorption of some drugs with poor oral bioavailability could be enhanced (Huang et al. 2018). Oridonin is a substrate of CYP3A4 and its transportation involves *P-gp*. Therefore, verapamil might make positive effect on the pharmacokinetics of oridonin. Although oridonin has been used clinically for a long time, there are little data available for the pharmacokinetics of oridonin, and the drug–drug interaction between oridonin and other drugs is lack of attention.

This study was focused on the effect of verapamil on the pharmacokinetics of oridonin in rats and clarified the main mechanism for its poor oral bioavailability. The *in vivo* pharmacokinetics of oridonin in rats with or without verapamil pre-treatment were determined using a sensitive and reliable LC-MS/MS method. Additionally, the effects of verapamil on the metabolism stability of oridonin were investigated in the rat liver microsomes and Caco-2 cell transwell model.

## Materials and methods

### Chemicals

Verapamil (purity >98%) and oridonin (purity >98%) was obtained from Shanghai Standard Biotechnology Co., Ltd. (Shanghai, China). Acetonitrile and methanol were purchased from Fisher Scientific (Fair Lawn, NJ, USA). Dulbecco’s modified Eagle’s medium (DMEM) and non-essential amino acid (NEAA) solution were purchased from Thermo Scientific Corp. (Logan, UT, USA). Foetal bovine serum (FBS) was obtained from GIBCO BRL (Grand Island, NY, USA). Penicillin G (10,000 U/mL) and streptomycin (10 mg/mL) were purchased from Amresco (Solon, OH, USA). Hanks’ balanced salt solution (HBSS) was purchased from GIBCO (Grand Island, NY, USA). Ultrapure water was prepared with a Milli-Q water purification system (Millipore, Billerica, MA, USA). All other chemicals were of analytical grade or better.

### Animal experiments

Male Sprague-Dawley rats weighing 230–250 g were provided by the experimental animal centre of the Jining Traditional Chinese Medicine Hospital. Rats were bred in a breeding room at 25 °C with 60 ± 5% humidity and a 12 h dark-light cycle. Tap water and normal chow were given *ad libitum*. All of the experimental animals were housed under the above conditions, for a three-day acclimation period and fasted overnight before the experiments. All experimental procedures and protocols were reviewed and approved by the Animal Care and Use Committee of Shanghai University of TCM and were in accordance with the National Institutes of Health guidelines regarding the principles of animal care.

### *In vivo* pharmacokinetic study

To evaluate the effects of verapamil on the pharmacokinetics of oridonin, the rats were divided into two groups of six animals each. The pre-treatment group was pre-treated with verapamil at a dose of 10 mg/kg/day (dissolved directly in normal saline containing 0.5% methylcellulose at a concentration of 2 mg/mL) for 7 days before the administration of oridonin. Next, oridonin were orally administered to rats by gavage at a dose of 20 mg/kg. The dose of verapamil and oridonin were based on previous reports (Xu et al. 2006; Zhu et al. 2017; Huang et al. 2018; Zhou et al. 2019). The control group was without the pre-treatment of verapamil before the oral administration of oridonin. Blood samples (250 µL) were collected into heparinized tubes via the *oculi chorioideae* vein at 0.083, 0.33, 0.5, 1, 2, 4, 6, 8, 10, 12, 24 and 36 h after the oral administration of oridonin. The blood samples were centrifuged at 3500 rpm for 5 min. The plasma samples that were obtained were stored at −40 °C until analysis.

### Preparation of rat plasma samples

To 100 µL aliquot of a plasma sample, 20 µL methanol and 180 µL internal standard methanol solution (2 ng/mL) were added and vortexed for 60 s to mix in a 1.5 mL polypropylene tube, and were centrifuged at 12,000 rpm for 10 min. The supernatant was removed into an injection vial, and a 3 µL aliquot was injected into the LC-MS/MS system for analysis.

### LC-MS/MS determination of oridonin

The determination of oridonin was performed on Agilent 1290 series liquid chromatography system and an Agilent 6470 triple-quadruple mass spectrometer (Palo Alto, CA, USA). The HPLC/MS conditions and sample preparation were basically according to a validated HPLC method described before (Jin et al. 2015; Zhang et al. 2019). The chromatographic analysis of oridonin was performed on a Waters X-Bridge C18 column (3.0 × 100 mm, i.d.; 3.5 µm, USA) at room temperature (25 °C). The mobile phase was water (containing 0.1% formic acid) and acetonitrile (30:70, v:v) with isocratic elution at a flow rate of 0.2 mL/min, and the analysis time was 4 min.

The mass scan mode was positive MRM mode. The precursor ion and product ion are *m/z* 365.3→347.3 for oridonin and *m/z* 252.1→155.9 for IS. The collision energy for oridonin and IS were 30 and 20 eV, respectively. The MS/MS conditions were optimized as follows: fragmentor, 110 V; capillary voltage, 3.5 kV; nozzle voltage, 500 V; nebulizer gas pressure (N_2_), 40 psig; drying gas flow (N_2_), 10 L/min; gas temperature, 350 °C; sheath gas temperature, 400 °C; sheath gas flow, 11 L/min.

### Cell culture

The Caco-2 cell line was obtained from the American Type Culture Collection (Manassas, VA, USA), and it was performed according to the previous study (Liu et al. 2018). The Caco-2 cells were cultured in DMEM high glucose medium containing 15% FBS, 1% NEAA and 100 U/mL penicillin and 100 µg/mL streptomycin. The cells were cultured at 37 °C with 5% CO_2_. For transport studies, the cells at passage 40 were seeded on transwell polycarbonate insert filters (1.12 cm^2^ surface, 0.4 µm pore size, 12 mm diameter; Corning Costar Corporation, MA, USA) in 12-well plates at a density of 1 × 10^5^ cells/cm^2^. Cells were allowed to grow for 21 days. For the first seven days, the medium was replaced every two days, and then daily. The transepithelial electrical resistance (TEER) of the monolayer cells was measured using Millicell ERS-2 (Millipore Corporation, Billerica, MA, USA), and TEER exceeding 400 Ω·cm^2^ was used for the flux experiment. The integrity of the Caco-2 monolayers was confirmed by the paracellular flux of Lucifer yellow, which was less than 1% per hour. The alkaline phosphatase activity was validated using an Alkaline Phosphatase Assay Kit. The qualified monolayers were used for transport studies.

### Effects of verapamil on the absorption of oridonin in the Caco-2 cell transwell model

Before the transport experiments, the cell monolayers were rinsed twice using warm (37 °C) HBSS, then the cells were incubated at 37 °C for 20 min. After preincubation, the cell monolayers were incubated with oridonin in fresh incubation medium added on either the apical or basolateral side for the indicated times at 37 °C. The volume of incubation medium on the apical and basolateral sides was 0.5 mL and 1.5 mL, respectively, and a 100 µL aliquot of the incubation solution was withdrawn at the indicated time points from the receiver compartment and replaced with the same volume of fresh pre-warmed HBSS buffer. The permeability of oridonin (2 µM) in all of the above condition s for both directions, i.e., from the apical (AP) side to the basolateral (BL) side and from the BL side to the AP side, was measured after incubation for 30, 60, 90 and 120 min at 37 °C. In addition, the efflux activity of *P-gp* was validated using a typical *P-gp* substrate digoxin (25 µM).

The apparent permeability coefficient (*P_app_*) was calculated using the equation of Artursson and Karlsson:

$$P_{\mathrm{app}}=\left(\Delta \text{Q}/\Delta \text{t}\right)\times \left[\text{1}/\left(\text{A}\times {\text{C}}_{0}\right)\right]$$

where *P_app_* is the apparent permeability coefficient (cm/s), Δ*Q*/Δ*t* (µmol/s) is the rate at which the compound appears in the receiver chamber, *C*_0_ (µmol/L) is the initial concentration of the compound in the donor chamber and A (cm^2^) represents the surface area of the cell monolayer. Data were collected from three separate experiments, and each was performed in triplicate. In addition, the efflux activity of *P-gp* was validated using a typical *P-gp* substrate digoxin (25 µM).

### Effects of verapamil on the metabolic stability of oridonin in rat liver microsomes

Rat liver microsomes were used to investigate the effects of verapamil on the metabolism clearance of oridonin, and the assay conditions and reaction mixtures were similar to those reported previously (Wang et al. 2017; Yan et al. 2017). In brief, 30 µL rat liver microsome (20 mg/mL), 12 µL oridonin solution (100 µM) and 1113 µL PBS buffer (0.1 M, pH 7.4) were added to the centrifuge tubes on ice. There was a 5 min preincubation step at 37 °C before initiating the reaction by adding NADPH-generating system (45 µL) into the microsomal suspension. The effects of verapamil or ketoconazole (a positive CYP3A4 inhibitor) on the metabolic stability of oridonin were investigated by adding 10 µM of verapamil or ketoconazole (12 µL, final concentration of 0.1 µM) to rat liver microsomes and preincubating them for 30 min at 37 °C, followed by the addition of NADPH-generating system. Aliquots of 100 µL were collected from the reaction volumes at 0.083, 0.167, 0.33, 0.5, 1, 2, 4, 8, 12, 24 and 36 h after the addition of oridonin, and 200 µL ice-cold acetonitrile containing esculin was added to terminate the reaction. All the experiments were performed in triplicate. The subsequent sample preparation method was same as the method of plasma sample preparation, and the concentration of oridonin was determined by LC-MS.

The *in vitro* half-life (*t*_1/2_) was obtained using the equation: *t*_1/2_=0.693/*k*; V (µL/mg)=volume of incubation (µL)/protein in the incubation (mg); Intrinsic clearance (Clint) (µL/min/mg protein)=V × 0.693/*t*_1/2_.

### Data analysis

The pharmacokinetic parameters, including the area under the plasma concentration-time curve (*AUC*), maximal plasma concentration (*C*_max_), the time for the maximal plasma concentration (*T*_max_), and the mean residence time (*MRT*) were calculated using the DAS 3.0 pharmacokinetic software (Chinese Pharmacological Association, Anhui, China).

The differences between the mean values were analysed for significance using a one-way analysis of variance (ANOVA). Values of *p* < 0.05 were considered to be statistically significant.

## Results

### Effect of verapamil on the pharmacokinetics of oridonin

**Figure 1.**
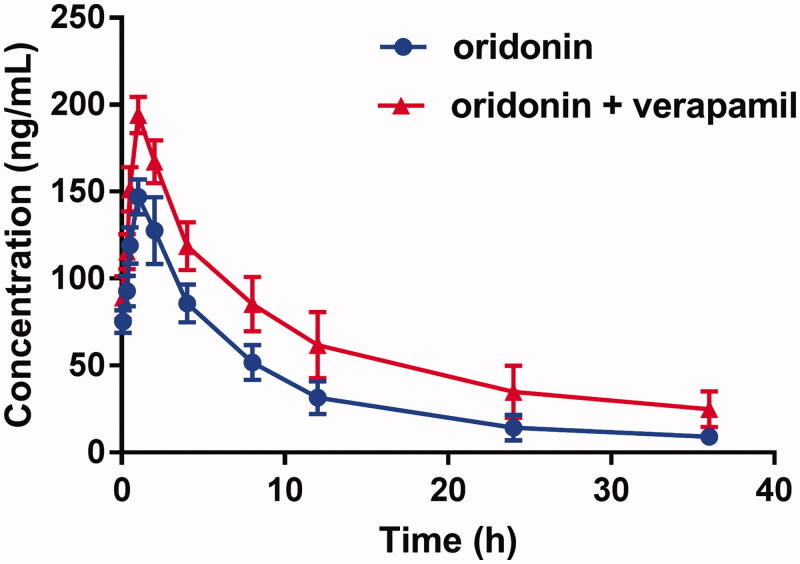
The pharmacokinetic profiles of oridonin in rats (six rats in each group) after the oral administration of 20 mg/kg oridonin with or without verapamil pre-treatment (10 mg/kg/day for 7 days). Each point represents the average ± SD of six determinations.

The mean plasma concentration-time curves of oridonin with or without verapamil are shown in Figure 1, and the pharmacokinetic parameters were calculated using the noncompartmental method with the DAS 3.0 pharmacokinetic software (Chinese Pharmacological Association, Anhui, China). The pharmacokinetic parameters are summarized in Table 1.

**Table 1.** Pharmacokinetic parameters of oridonin in rats after intragastrical administration of oridonin (40 mg/kg; *n* = 6, mean ± SD) with or without treatment of verapamil.

Parameter	Control	Pre-treatment of verapamil
*T*_max_ (h)	1.00 ± 0.12	1.00 ± 0.18
*C*_max_ (ng/mL)	146.9 ± 10.17	194.0 ± 10.53*
*t*_1/2_ (h)	10.88 ± 4.38	13.69 ± 4.81*
AUC_(0–_*_t_*_)_ (mg·h/L)	1.31 ± 0.29	2.23 ± 0.53*
MRT (h)	9.25 ± 1.10	11.48 ± 1.50*
CLz/F (L/h/kg)	14.69 ± 4.42	8.09 ± 3.03*

* *p* < 0.05 indicates significant differences from the control.

As shown in Table 1, after the pre-treatment with verapamil, the peak plasma concentration (*C*_max_) of oridonin increased from 146.9 ± 10.17 to 193.97 ± 10.53 ng/mL, and the difference was significant (*p* < 0.05). In addition, verapamil significantly increased the AUC_0–_*_t_* of oridonin (1.31 ± 0.29 vs. 2.23 ± 0.53 mg·h·L^−1^, *p* < 0.05). Both results indicated the administration of verapamil could improve the concentration of oridonin in the blood plasma. In the meantime, the half-life (*t*_1/2_) and the mean residence time (*MRT*) of oridonin prolonged with the preincubation of verapamil, and the clearance rate declined. These results suggested that verapamil increased the plasma concentration and the system exposure of oridonin.

### Effects of verapamil on the bidirectional transport of oridonin across Caco-2 cells

**Figure 2.**
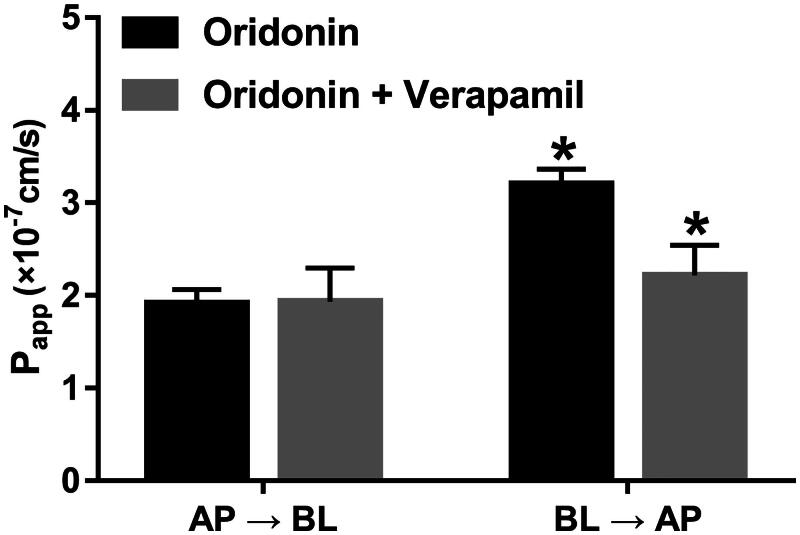
Effects of verapamil on the transport of oridonin from the apical to basolateral side or the opposite direction, Caco-2 cell monolayers were incubated at 37 °C in HBSS (pH 7.4), and oridonin (2 µM) were added to the apical or basolateral side, verapamil were also added to the donor chamber with oridonin. *Significant differences (*p* < 0.05) were seen compared to the control sample. Each point represents the mean ± SD of 3 determinations.

The effect of verapamil on the transport of oridonin was studied via the Caco-2 cells *in vitro* model. Digoxin a typical *P-gp* substrate was employed to validate the efflux activity of *P-gp*. The result showed the efflux ratio of digoxin was 10.12 and it was abrogated when verapamil was present. The results indicated that the efflux activity of *P-gp* was qualified for the experiment. As shown in Figure 2, the *P_appAB_* and *P_appBA_* of oridonin were 1.91 ± 0.15 × 10^−7^ and 3.20 ± 0.16 × 10^−7 ^cm/s, respectively, and the efflux ratio was 1.67. The result showed that *P_appBA_* was much higher than *P_appAB_*, which indicated that efflux transporters might be involved in the transport of oridonin. Verapamil decreased the *P_appBA_* of oridonin to 2.22 ± 0.32 × 10^−7 ^cm/s, and the efflux ratio decreased to 1.15. These results indicated that the efflux of oridonin was inhibited when verapamil was present.

### Effects of verapamil on the metabolic stability of oridonin in rat liver microsomes

The effect of verapamil on the metabolic stability of oridonin was investigated in rat liver microsomes. During the incubation, the metabolic half-life (*t*_1/2_) of oridonin was 34.6 ± 2.7 min, and it prolonged to 38.4 ± 3.3 min in the presence of verapamil, the difference was significant (*p* < 0.05). Meanwhile, the intrinsic clearance rate of oridonin decreased from 40.06 ± 2.5 to 36.09 ± 3.7 µL/min/mg protein, when verapamil was present. It suggested that verapamil inhibited the metabolism of oridonin in rat liver microsomes, and decreased the intrinsic clearance rate of oridonin at the same time. These results also verified the results of pharmacokinetics study.

## Discussion

It has been reported that oridonin has the ability of inhibiting various cancers. However, due to its poor bioavailability, the therapeutic uses of oridonin was limited. Verapamil can enhance the absorption of the drugs with poor bioavailability, via inhibiting the activity of CYP3A4 and *P-gp* (Guo et al. 2018; Huang et al. 2018; Zhou et al. 2019). Whether verapamil has the same effect on oridonin is still unknown. As co-administration of different drugs has become more and more normal in clinic, the drug–drug interaction between verapamil and oridonin needs attention. The effect of verapamil on the pharmacokinetics of oridonin in rats was investigated in the present study, and the main mechanism was clarified. The results indicated that verapamil increased the system exposure of oridonin, as the *C*_max_ and AUC_(0–_*_t_*_)_ increased with the pre-treatment of verapamil. Additionally, verapamil decreased the *t*_1/2_ and oral clearance of oridonin, which indicated verapamil made inhibitory effect on the metabolism of oridonin. Meanwhile, the rat liver microsomes were employed to investigate the effect of verapamil on the metabolic stability of oridonin. In rat liver microsomes, the metabolism of oridonin was inhibited by verapamil, and the intrinsic clearance rate decreased. According to the previous studies, verapamil acts an inhibitor of CYP3A4 (Piao et al. 2008; Zhu et al. 2017; Chen et al. 2018), and CYP3A4 also participates in the metabolism of oridonin (Xu et al. 2006), we inferred that verapamil inhibited the metabolism of oridonin through inhibiting the activity of CYP3A4.

Efflux and absorption also play vital roles in the bioavailability of drugs. The Caco-2 cell monolayer model was used to explore the effect of verapamil on the absorption and efflux of oridonin. As shown from the results, the efflux of oridonin was much higher than the influx, which indicated *P-gp* might be involved in the transport of oridonin. Verapamil decreased the efflux ratio of oridonin, and the absorption of oridonin was enhanced. Verapamil has been reported as an inhibitor of *P-gp*, which exerts the effects of pumping oridonin out of cells (Zhu et al. 2017). We concluded that verapamil accelerates the absorption of oridonin via inhibiting the activity of *P-gp*. Moreover, there are various types of CYP enzymes, previous studies and the present study mainly focused on the function of CYP3A4 in the inhibitory effect of verapamil. Whether verapamil can induce the drug–drug interactions through the effect on other CYP isoforms needs further investigation.

In summary, the co-administration of oridonin and verapamil affected the pharmacokinetics and transport of oridonin. As shown from the above results, due to the inhibitory effect of verapamil on the activity of CYP3A4 and *P-gp*, verapamil inhibited the metabolism and efflux of oridonin, as the *C*_max_ and AUC_(0_*_–t_*_)_ of oridonin increased and the *t*_1/2_ prolonged. However, the solubility and dissolution rate also influence the bioavailability of drugs, and therefore requires additional investigation.

## Conclusions

Verapamil increased the system exposure of oridonin via inhibiting the activity of CYP3A4, which is closely related to the metabolism of oridonin. On the other hand, verapamil inhibited the activity of *P-gp*, and then inhibited the efflux of oridonin and enhanced its absorption. Concomitant verapamil and oridonin treatment would allow dose reduction and still achieve comparable exposure of oridonin.
